# The impact of using fresh frozen plasma in cardiopulmonary bypass preparation on thromboelastometric parameters and receiving blood products among pediatric patients undergoing cardiac surgery

**DOI:** 10.34172/jcvtr.2023.30511

**Published:** 2023-03-16

**Authors:** Maryam Abedzadeh, Naser Kachoueian, Azadeh Fazli, Maryam Pazhoha, Samira Orouji Omid, Parvin Vahid, Nader Givtaj

**Affiliations:** ^1^Rajaie Cardiovascular Medical and Research Center, Iran University of Medical Sciences, Tehran, Iran; ^2^Department of Cardiac Surgery, Imam Hossein Educational Hospital, Shahid Beheshti University of Medical Sciences, Tehran, Iran; ^3^Azad University of Medical Sciences, Tehran, Iran; ^4^Mazandaran University of Medical Sciences, Mazandaran, Iran

**Keywords:** Fresh Frozen Plasma (FFP), Priming, ROTEM, Cardiopulmonary Bypass

## Abstract

***Introduction:*** The aim of this study was to determine the effect of fresh frozen plasma (FFP) for priming of cardiopulmonary bypass (CPB) circuit on rotational thromboelastometry (ROTEM) and transfusion in pediatric cardiac surgery.

***Methods:*** Eighty patients younger than seven years old, were divided into case (FFP) (n=40) and control (n=40) groups. In the case group,10-20 mL/kg fresh frozen plasm was used for priming the CPB. The control group received 10-20 mL/kg of hydroxyethyl starch. ROTEM was done before surgical incision and after separation from CPB. The amount of transfusion (platelet and FFP) in the operating room and 24 hours after surgery were recorded.

***Results:*** Statistically significant difference was found between the case and control group in terms of changes in the Rotem parameters. The amount of transfusion of platelets in the operating room was significantly higher in the control group than in the case group.

***Conclusion:*** It seems that adding FFP to the prime solution is more effective in young patients and infants due to the higher susceptibility of the infant coagulation system to coagulation and hemorrhagic disorders in comparison with other patients.

## Introduction

 Annually worldwide, around 400 000 open-heart surgeries are performed using cardiopulmonary bypass system, with about 4% of these cases claimed by pediatric cardiac surgery.^1^ Different studies had been performed in most of one-pomp cardiac surgery related field such as surgical stress response, blood transfusion requirement, inflammatory markers, hypothermia, and coagulation cascades.^2-7^ Among them, severe bleeding in on-pump cardiac surgery has been considered as the most important cause of complications and mortality. A prospective study indicated that during the operation, administration of blood, albumin, and fresh plasma, and postoperative administration of platelets have been higher among patients undergoing surgery with cardiopulmonary bypass system compared to other patients.^8^ In addition, issues such as mechanical damage resulting from direct manipulation of blood vessels by the surgeon, dilutional coagulopathy resulting from use of crystalloid solutions and colloidal solutions priming of the cardiopulmonary bypass system, or consumptive coagulopathy and fibrinolysis have been reported as the causes of complications of employing this machine.^9^

 The results of different studies have indicated that the main cause of coagulopathy and bleeding during and after on-pump cardiac surgery is use of different solutions for preparation of this system. In some cases, these solutions can even cause 35% reduction in the proteins of coagulation and fibrinolytic factors in patients. However, stimulation of coagulation and fibrinolysis pathways reduce coagulation factors to only 1%.^10^

 Ideal solution for priming of cardiopulmonary bypass (CPB) is still a challenging issue in cardiac surgery. In the past, the preparation solutions were solutions with electrolyte content and osmolarity like intravascular fluids, so that once mixed with the blood, the oxygenation, carbon dioxide expulsion, and physiologic homeostasis of patients would be maintained. From the mid-20th century, use of blood dilution during cardiac operation was studied, and extensive research has been performed to find a suitable solution for preparing cardiopulmonary bypass system. The most important effect of blood dilution in this system especially in children is the negative effects on coagulation system occurring due to severe reduction in the concentration of coagulation factors.^11^ Currently, in order to minimize the volume of the solution utilized for preparation of the cardiopulmonary bypass system, various approaches are used including removing parts of the circuit such as the artery filters. Nevertheless, this may threaten the patients’ safety.^12^ Because dilutional coagulopathy is affected by the type of solution utilized for preparing the cardiopulmonary bypass system,^13^ the present research postulates that usage of fresh frozen plasma in the preparation stage of cardiopulmonary bypass system could prevent dilutional coagulopathy during the bypass. Accordingly, the aim of the present study is to determine the effect of use of fresh frozen plasma in the preparation stage of cardiopulmonary bypass system on the thromboelastometric parameters and administration of blood products among children undergoing cardiac surgery.

## Materials and Methods

 This randomized clinical trial was performed on children < 7 years with congenital heart disease in Shahid Rajaei Cardiovascular, Medical Research Center. The protocol of this study was approved by the ethics committee of Iran University of medical sciences with the code of IRIUMS.REC.1395.9211564202. Inclusion criteria include: age < 7 years with no previous cardiac surgery, coagulation or hepatic disorders. Among the participants, the patients who died during the preoperative period or had transfusion of blood product over the past 24 hours were excluded. Based on the studied parameters (α = 0.05, β = 0.2, d = 50), the sample size required for the study was estimated 40 patients in each group. Random allocation method was used in order to allocate the patients in either the case (FFP) or control groups.

 Induction and maintenance of anesthesia were the same in both groups. For all patients, in the operation room, routine monitoring in pediatric cardiac surgery include: ECG, IBP, CVP, B.T, Pulse oximetry and cerebral oximetry were recorded. Before surgical incision, coagulation function of both groups were investigated through rotational thromboelastometry (ROTEM) including two pathway: INTEM and EXTEM.

 For priming of the cardiopulmonary bypass (CPB), in case group, 10-20cc/kg FFP plus 200-300 ml crystalloid solution (Ringer) and in control group, 10-20 cc/kg hydroxyethyl starch with 130 to 0.4 ratio plus 100-200 ml crystalloid solution (Ringer) were used. During the bypass, Hct was maintained at 25-30% and the mean arterial pressure within 40-70 mm Hg considering age and temperature. After weaning from the CPB and heparin reversal, another ROTEM including two pathway: INTEM and EXTEM was done. Amount of transfusion (platelets, packed red blood cells, fresh frozen plasma, and Cryoprecipitate) during operation and first postoperative day was recorded.

###  Statistical analysis

 In this study, the obtained data were introduced into SPSS for analysis. For descriptive analysis of the quantitative data, mean and standard deviation, while for qualitative data, frequency and percentage were used. In this study, for data analysis, chi-square test was employed for quantitative data, while independent sample t-test for quantitative data. All results of statistical tests were significant with *P* < 0.05.

## Results

 In this study 80 children younger than seven years old age who were candidates for cardiac surgery assigned into FFP and control groups (n = 40 in each) were evaluated. Findings of comparing demographic and basal variables between FFP and control groups were presented in Table 1. According that, the participants of both groups were homogeneous in terms of age and gender, and the mean age as well as gender frequency of the children between the two groups did not have significant differences with each other considering the parameters associated with surgery, the mean cardiopulmonary bypass time in the children examined in the two groups did not differ significantly (74.57 ± 21.54 vs. 87.27 ± 38.20 min; *P* = 0.07). Although, two (5%) patients in FFP and 6 (15%) patients in control group had cyanotic disorders, there was no significant difference for frequency of cyanotic disorders between two groups (*P* = 0.26). In contrast, the aortic cross clamp time in FFP group was significantly longer (30.10 ± 17.45 vs. 19.07 ± 24.33 min; *P* = 0.02).

**Table 1:** comparing demographic variables between two trial groups

**Study groups Variables**	**FFP (n=40)**	**Control (n=40)**	***P*** ** value**
Gender (male)	40 (22, 55%)	40 (21, 52.5%)	0.83
Age (months)	20.6 ± 13.49	27.75 ± 20.06	0.07
Height (cm)	78.48 ± 12.88	80.72 ± 15.22	0.48
Weight (Kg)	8.80 ± 2.79	9.48 ± 3.14	0.31
BSA	0.44 ± 0.11	0.47 ± 0.12	0.29
Cyanotic disorders	2 (5%)	6 (15%)	0.26
Temperature	31.55 ± 1.50	32.19 ± 1.62	0.072
CBT	74.57 ± 21.54	87.27 ± 38.21	0.071
Clamping time	30.1 ± 17.46	19.08 ± 24.24	0.022

Abbreviations: BSA, body surface area; CBT, Cardiopulmonary bypass time; FFP, fresh frozen plasma

###  ROTEM before heparin and surgical incision

 In the EXTEM and INTERM, none of parameters did not have significant difference between patients of FFP and control groups.

###  ROTEM after weaning from CPB

 In the INTEM, the mean CT and CFT were significantly higher among the FFP group subjects compared to the control (*P* = 0.03). The mean of alpha angle was significantly higher in the control group subjects than FFP group subjects (*P* = 0.04). In the EXTEM, only mean of alpha angle were significantly higher in the FFP group subjects compared to the control (*P* = 0.001). Other parameters had not significant differences between study subjects in both groups. ROTERM parameters before heparin and surgical incision were presented in Table 2 and ROTERM parameters after weaning from CBP were presented in Table 3.

**Table 2:** ROTEM before heparin and surgical incision

**Groups** **Thromboelastometry** **Parameters**	**Pathway**	**Trial group (n=40)**	**Control group (n=40)**	***P*** ** value**
		**Mean±SD**	**Mean±SD**	
CT (s)	EXTEM	76.12 ± 38.44	71.85 ± 38.30	0.62
CFT (s)	EXTEM	94.25 ± 69.78	98.33 ± 44.93	0.76
(Alpha Angle °)	EXTEM	71.93 ± 8.27	69.60 ± 8.88	0.23
MCF (mm)	EXTEM	58.05 ± 9.82	54.90 ± 15.15	0.27
CT (s)	INTEM	249.35 ± 91.52	260.54 ± 88.30	0.75
CFT (s)	INTEM	97.83 ± 60.42	143.64 ± 68.21	0.11
Alpha angle (°)	INTEM	72.68 ± 7.62	71.35 ± 14.19	0.61
MCF (mm)	INTEM	53.72 ± 10.18	49.48 ± 14.87	0.15

Abbreviations: CT, clotting time; CFT, clot formation time; MCF, maximum clot firmness

**Table 3:** ROTEM after weaning from CPB and heparin reversal

**Groups** **Thromboelastometry** **Parameters**	**Pathway**	**Trial group (n=40)**	**Control group (n=40)**	***P*** ** value**
		**Mean±SD**	**Mean±SD**	
CT (s)	INTEM	253.81 ± 70.02	325.67 ± 157.92	0.03
CFT (s)	INTEM	170.97 ± 111.01	234.14 ± 139.40	0.03
Alpha angle (°)	INTEM	62.65 ± 10.10	52.40 ± 19.10	0.04
CT (s)	EXTEM	90.23 ± 51.69	96.75 ± 84.69	0.47
CFT (s)	EXTEM	166.29 ± 116.28	230.60 ± 153.99	0.05
Alpha angle (°)	EXTEM	63.63 ± 13.93	32.80 ± 18.17	0.001
MCF (mm)	EXTEM	44.71 ± 11.63	37.02 ± 15.89	0.18

Abbreviations: CT, clotting time; CFT, clot formation time; MCF, maximum clot firmness; ML;maximum lysis.

 According to findings that were present in Table 4, in the first postoperative day, although, transfusion of FFP, PRBC, cryoprecipitate in operation room had not significant differences between patients in FFP and control groups, the platelet transfusion in operation room was significantly higher in the control group in compare with FFP group (*P* = 0.03).

**Table 4:** Amount of transfusion in the operation room and first postoperative day

**Groups Blood product Receiving**	**FFP (n=40)**	**Control (n=40)**	***P*** ** value**
	**Mean±SD**	**Mean±SD**	
PC in Prime (cc)	71.48 ± 53.97	94.75 ± 54.35	0.06
PC infusion OP (cc)	88.89 ± 76.31	95.50 ± 73.83	0.70
FFP Infusion OP (cc)	11.65 ± 33.39	5.01 ± 31.62	0.36
Fibrinogen infusion OP (mg)	39 ± 133.28	1.02 ± 6.32	0.08
PC infusion ICU (cc)	104.67 ± 81.08	99.02 ± 74.73	0.75
FFP Infusion ICU (cc)	41 ± 77.38	61.75 ± 95.18	0.28
Platelets (cc)	3.08 ± 13.41	7.50 ± 26.67	0.03

Abbreviations: OR, operation room; ICU, intensive care unit; FFP, fresh frozen plasma; PC, packed cell; OP, operation

## Discussion

 The present study was performed to evaluate the effect of using of fresh frozen plasma for priming of cardiopulmonary bypass on thromboelastometric parameters and transfusion of blood products in the operation room and ICU. Fresh Frozen Plasma (FFP) is one of the recent substances which is used for priming the CPB circuit. However, in several randomized clinical trials, investigators had challenge with safety and prophylactic priming of CPB with FFP.^14-16^ Dieu et al reported that in infants and small children, priming CPB circuit with crystalloids is not different compared with priming based on FFP in terms of postoperative blood loss and transfusion of allogeneic blood products.^17^

 In the INTEM and before surgical incision and heparin administration, none of permeates had not significant difference between two groups. In the internal and external pathways after injection of protamine and getting disconnected from the cardiopulmonary bypass machine, only alpha angle and CFT had significantly higher in subjects of FFP group in compare with control group. Various reasons can explain this coagulopathy followed by bleeding and transfusion of blood products such as consumption of coagulation factors during the cardiopulmonary bypass, stimulation of the inflammatory system, and blood dilution during the cardiopulmonary bypass. In a similar study by Yang in 2013 to compare the effects of fresh frozen plasma and albumin among children and infants on changes in thromboelastometric parameters plus transfusion of blood products, it was found that the studied patients had shorter CFT and higher alpha angle in the internal and external pathways after injection of protamine. A10, MCF were higher in both children and infants’ groups in both INTEM and EXTEM paths in the group receiving fresh frozen plasma. Nevertheless, in first postoperative day, these changes were not significant. Indeed, it can be stated that adding fresh frozen plasma to the cardiopulmonary bypass reduces the incidence of coagulopathy following the bypass. This can well be observed in changes occurring in CT, CFT, and MCF parameters among both children and infants. Nevertheless, this beneficial effect of fresh frozen plasma in the priming of the cardiopulmonary bypass circuit persists only up to 24 hours post-surgery.^8^ The results of the mentioned study have contrasted with ours, since in this study the two groups did not differ significantly. Nevertheless, in the present study, hydroxyethyl starch 6% and fresh frozen plasma were compared, but in the study by Despotis et al albumin and fresh frozen plasma were investigated. In addition, in their study, infants and children group were separated and investigated at three times. In another study by Juachim in 2009, it was found that thromboelastometric parameters were less affected in the group receiving hydroxy ethyl starch compared to the group receiving albumin. CFT and MCF where similar and normal in both groups. Following cardiac surgery, CT and CFT increased compared to the baseline, with CFT and CT being larger in the albumin group compared to the hydroxy ethyl starch group. MCF was similar in both groups while the extent of platelet agglutination remained unchanged in the hydroxy ethyl starch group, and the extent of agglutination decreased in the albumin group. The results of this study indicated that starch maintains the platelet function better than albumin does.^9^ In a study performed to compare the effects of use of albumin 5% and hydroxy ethyl starch in the preparation stage of cardiopulmonary bypass machine on the renal function and coagulation status of patients undergoing coronary surgery, the researchers found that the coagulation status and renal function of the patients were maintained better in the group receiving albumin compared to hydroxy ethyl starch. According to their analysis, albumin coats the surfaces of the cardiopulmonary bypass circuit like an endothelium membrane, and prevents platelet agglutination to the mentioned surfaces.^10^ In another study performed in Myoclonic in America, the effect of applying albumin 5% and fresh frozen plasma at the preparation stage of cardiopulmonary pump was explored on coagulation tests and administration of blood products following cardiopulmonary bypass surgery in children with weight lighter than 10 kg. The results of that study showed that 10 minutes following protamine administration, the patients who had received fresh frozen plasma had shorter prothrombin time, but the level of fibrinogen was significantly higher. Nevertheless, the extent of activated clotting time, prothrombin time, relative prothrombin, and platelet count did not differ significantly between the two groups in the early hours in the ICU. In addition, the results of this study indicated that the type of solution used for preparing the cardiopulmonary pump as well as its impact on coagulation factors depend on the type of cardiac surgery (simple or complicated). Accordingly, the researchers propose that in patients with non-cyanotic cardiac disease undergoing simple surgery, albumin 5% is a suitable substitute for fresh frozen plasma. Nevertheless, for cyanotic patients who undergo complex surgery, further investigations are required.^11^

 A meta-analysis to explore the effects of different types of hydroxyethyl starch on bleeding following cardiopulmonary bypass on 18 clinical trials with 970 patients indicated that bleeding increased by up to 33.3% with hydroxyethyl starch in comparison to albumin, and the risk of re-surgery was twice as large in the group receiving hydroxy ethyl starch compared to the albumin receivers because of bleeding. The results of this study showed that albumin improved the hemodynamic status of patients, while different types of hydroxy ethyl starch would increase the level of bleeding in different ways. Investigations performed through thromboelastometry showed that these solutions would reduce the maximum clot resistance, but there is no specific information about hydroxy ethyl starch with molecular weight lower than 130 to 0.4 and albumin.^12^

 Abbaszadeh Ghanavati et al in their study concluded that the extent of receiving blood products such as fresh frozen plasma was higher in the test group compared to the control considering the amount utilized in the preparation stage of cardiopulmonary bypass machine among children and infants, while the level of infusion of packed red blood cells was the same in both groups.^8^ Balajudia in his study reported that use of fresh frozen plasma in the preparation stage of the cardiopulmonary bypass machine would reduce post operation bleeding among children younger than six months of age. Thus, the need to administer blood products in them would decrease.^13^

 In another study, the effects of fresh frozen plasma were investigated in the preparation stage of the cardiopulmonary bypass. It was found that adding fresh frozen plasma in the preparation stage would reduce hypofibrinogenemia.^5^ Different showed that the most important dilutional coagulopathy following cardiopulmonary bypass surgery is hypofibrinogenemia, and use of fresh frozen plasma can reduce its incidence of to 24 hours after this surgery. In the present study, fibrinogen in patients cannot be judged since other thromboelastometric paths were investigated. Thus, according to the information available about the internal and external pathways, only one disorder can be diagnosed, though its details require further investigation. Investigation of the present study and other studies conducted in this area shows that use of fresh frozen plasma in the preparation stage of the cardiopulmonary bypass machine as a colloidal solution among children causes maintenance of the clot resistance and improving coagulation factors. As such, they have less need to receive blood products especially platelet during the operation. Nevertheless, the extent of blood product administration 24 hours after hospitalization in the ICU did not differ significantly between the two groups. Considering the executive limitations of this study regarding the collection of data required for more extensive analysis, it is suggested that future studies explore the effect of use of fresh frozen plasma on inflammatory factors, temperature patients in ICU, and the amount of blood product administration in patients suffering from cyanotic cardiac diseases.

## Conclusion

 In against impairment in thromboelastography parameters in control group, transfusion of blood and blood products had not significant difference between two groups. It seems that adding FFP to the prime solution is more effective in young patients and infants due to the higher susceptibility of the infant coagulation system to coagulation and hemorrhagic disorders in comparison with other patients.

## Acknowledgements

 Authors gratitude all of persons that help them to study design and statistical analysis.

## Competing Interests

 Authors did not have any conflict of interest in this manuscript.

## Ethical Approval

 The protocol of this study was approved by the ethics committee of Iran University of medical sciences with the code of IRIUMS.REC.1395.9211564202.

## Funding

 Authors did not any special fund for performing this study.
